# Sex Disparity in Severity of Lung Lesions in Newly Identified Tuberculosis Is Age-Associated

**DOI:** 10.3389/fmed.2019.00163

**Published:** 2019-07-17

**Authors:** Yue Chu, Adiilah K. Soodeen-Lalloo, Jin Huang, Guanghong Yang, Fengfang Chen, Hongyun Yin, Wei Sha, Xiaochen Huang, Jingyun Shi, Yonghong Feng

**Affiliations:** ^1^Shanghai Key Laboratory of Tuberculosis, School of Medicine, Clinical and Research Centre of Tuberculosis, Shanghai Pulmonary Hospital, Tongji University, Shanghai, China; ^2^Department of Radiology, School of Medicine, Shanghai Pulmonary Hospital, Tongji University, Shanghai, China; ^3^Key Laboratory of Environment Pollution Monitoring and Disease Control, School of Public Health, Guizhou Medical University, Ministry of Education, Guiyang, China

**Keywords:** tuberculosis, sex bias, computed tomography, complement C4, BMI

## Abstract

**Background:** The age-associated characteristic of computed tomography (CT) images of tuberculosis (TB) and the reason for male bias in TB are still not clear.

**Methods:** We compared the CT images, clinical inflammatory indices and sputum bacterial counts between 594 non-smoking men and women with newly diagnosed TB with matched large span of ages from 15 to 92 years old. Logistic regression analyses were used to identify the cavity-associated factors of men and women, separately and in combination.

**Results:** Sputum bacterial counts, ratio of cavities, lung injury scores, and level of C reactive protein were significantly higher in men than in women with ages from 15 to 74, but not in cases older than 75. In CT images, thick walled cavity, cicatricial emphysema and parenchymal bands were present in men at ages of 15–74 more than matched women. Ratios of cases with lobular emphysema and pleural effusion were higher in men after age of 56. While ratios of cases with parenchymal bands, calcification, pleural effusion, pleural thickening, lobular emphysema and bronchovascular distortion increased with aging, those of centrilobular nodules, micronodules and tree in bud decreased with aging in men. Erythrocyte sedimentation rate (ESR) increased with aging, but no differences were found between men and women in ESR or T-SPOT TB tests. Higher complement C4 and lower body mass index in men and positive result in anti-TB antibody test in women were strongly associated with the presence of cavity.

**Conclusions:** The sex bias in TB is age-associated. TB prevention, treatment and research should take differences of sex and age into account.

## Background

Tuberculosis (TB), which is induced by *Mycobacterium tuberculosis (Mtb*.), resulted in approximately 1.3 million deaths in 2017 and remains one of the top 10 causes of death and the leading cause of a single infectious agent worldwide (1). A male bias in case notification was noticed with a man to woman ratio of TB cases of around 1.5–2.1 in all regions of the world (1, 2), and the influence of different levels of accessibility to healthcare services between men and women has been excluded in a two-stage random sampling population survey through interviews of men and women in pairs (3).

In the human population, the gap between male and female reported TB cases seems to start after puberty (1) (2017-annext II). Consistently, the exacerbated pulmonary pathology and increased morbidity and mortality in male mice can be prevented by castration (4, 5). These observations indicated that age-associated factors, e.g., changed levels of sex hormones, may play key roles in pathogenesis of TB, and testosterone could be a TB susceptibility factor. Except for sex hormones, however, sex–related genetic background and regulation, and sex-specific metabolic features are also suggested to be correlated with the sex bias in TB pathogenesis (6–9). The underlying mechanisms of the sex bias in TB are still uncertain.

Sex hormone-associated differences in both the immune and the endocrine systems are influenced greatly by aging (10). In this study, we hypothesize that, through comparison of the lung lesions with computed tomography (CT) images and inflammatory indices between new cases of well-matched men and women with TB over a large span of ages (adolescent and adult, middle aged and gerontic aged), we can get clues for the interplay of sex-associated endocrines with the aging immune system on sex bias of TB pathogenesis.

## Methods

### Study Subjects

We included in a total of 594 newly diagnosed pulmonary TB (299 men, 295 women) with large span of ages (15 to 92 years old) from Shanghai Pulmonary Hospital (SPH) between Feb. 2012 and Nov. 2018. TB was diagnosed based on acid-fast bacilli (AFB) staining and culture; patients whose cultures yielded non-tuberculous mycobacteria were excluded from the study. We retrospectively reviewed medical records and excluded patients who had a history of any of the following: smoking, excessive alcohol drinking, human immunodeficiency virus infection, immunosuppressive drug therapy, hormone therapy, cancer, diabetes, pneumoconiosis, silicosis and Hepatitis B and C viruses infection (11). None of the patients had received anti-TB therapy for more than 1 week before registration. All the enrolled female (F) and male (M) TB subjects were matched for age (±3 years) and were classified according to their ages: adolescents and adults TB (F-TB_15−55_ and M-TB_15−55_, footnotes represent the range of ages), middle aged and early elderly TB (F-TB_56−74_ and M-TB_56−74_), and gerontic aged patients (F-TB_75−92_ and M-TB_75−92_).

### Review of Clinical Findings and Laboratory Tests

Routine inflammatory, hematological and biochemical parameters were reviewed. Grading of AFB, T-SPOT.TB tests and anti-TB Ig G antibody (TBAb) detection were carried out according to previous description (11).

### Computed Tomography Evaluation

In all 594 patients, high-resolution CT scans from 223 female and 223 male patients performed within the first week of admittance were collected and evaluated as in a previous report (11). In brief, lungs were divided into 6 zones (low, middle and high zones for left and right lungs) and the presence of the abnormalities including nodule, micronodule, cavity, consolidation, parenchymal bands, ground glass opacity and bronchial lesion were noted (12–15). The scans were assessed by two specialists who were blinded to the groups of patients. The total weighted profusion score was calculated as profusion score ×100 / 24 (total score) +40 if cavitation was present.

### Ethical Approval

This study was conducted in accordance with the amended Declaration of Helsinki and the ethical guidelines of the institutional review board of Tongji University (Project approval number: K17-043). All participants gave written consent for the use of their clinical information for research purposes. Clinical data were anonymized.

### Statistical Analyses

We performed χ^2^ test for categorical variables, Wilcoxon rank sum test for nominal variables, and *t* tests for continuous variables. To identify the parameters associated with the extent of lung lesions (cavity), 53 physiological, hematological and biochemical indices were involved in multivariate logistic regression with males and females analyzed separately and in combination. Statistical significance was determined at *p* < 0.05. All analyses were performed using SPSS (version 19, SPSS Inc., Chicago, IL, USA).

## Results

### Patient Characteristics

The physiological characteristics of the age-matched groups of men and women with TB are shown in Table 1. The paired groups showed no significant differences in BMI at the time of their first registration in SPH.

**Table 1:** Characteristics of study patients.

	**15–55**			**56–74**			**75–92**		
	**F-TB** **(*n =* 142)**	**M-TB** **(*n =* 145)**	***P-*value**	**F-TB** **(*n =* 103)**	**M-TB** **(*n =* 104)**	***P-*value**	**F-TB** **(*n =* 50)**	**M-TB** **(*n =* 50)**	***P-*value**
Age, year^†^	24 (18–32)	23 (18–32)	0.786^a^	65 (62–68)	65 (62–68)	0.959^a^	81 (78–85)	80 (78–84)	0.784^a^
Height, cm^†^	161 (159–165) *n =* 132	174 (170-178) *n =* 132	**<0.001**^a^	158 (152–160) *n =* 79	170 (165–172) *n =* 83	**<0.001**^a^	155 (152–159) *n =* 38	171 (167–175) *n =* 34	**<0.001**^a^
Weight, kg^†^	50 (45–55) *n =* 132	60 (54–65) *n =* 133	**<0.001**^a^	51 (43–56) *n =* 81	60 (52–64) *n =* 84	**<0.001**^a^	45 (40–55) *n =* 38	55 (49–60) *n =* 37	**<0.001**^a^
BMI, kg/m^2^^†^	19.1 (17.8–20.6) *n =* 132	19.6 (18.0–21.5) *n =* 132	0.433^a^	20.2 (18.2–23.1) *n =* 79	20.2 (18.6–22.1) *n =* 83	0.905^a^	19.1 (15.6–19.1) *n =* 38	19.3 (17.4–21.7) *n =* 34	0.757^a^
**Time elapsed between onset of symptoms and admittance to SPH, months, number (%)^1^**			**0.003^b^**			**0.007**^b^			0.406^b^
No–symptoms^2^	7 (4.9)	13 (9.0)	–	4 (3.9)	7(6.7)	–	0 (0.0)	3 (6.0)	–
<1	35 (24.7)	54 (37.2)	–	21 (20.4)	41 (39.4)	–	10 (20.0)	13 (26.0)	–
1–6	80 (56.3)	65 (44.8)	–	38 (36.9)	28 (27.0)	–	16 (32.0)	12 (24.0)	–
6–12	12 (8.5)	9 (6.2)	–	17(16.5)	8 (7.7)	–	7 (14.0)	5 (10.0)	–
>12	8 (5.6)	4 (2.8)	–	23 (22.3)	20 (19.2)	–	17 (34.0)	17 (34.0)	–
**T-SPOT.TB (+), number (%)**							
	73 (87.9) *n =* 83	83 (89.3) *n =* 93	0.787^c^	51 (92.7) *n =* 55	62 (91.2) *n =* 68	0.754^c^	16 (84.2) *n =* 19	17 (89.5) *n =* 19	0.631^c^

*TB, tuberculosis; BMI, body mass index; F-TB, female TB patients; M-TB, male TB patients*.

† *Data are displayed as medians and interquartile ranges*.

a *t-tests*.

b *Wilcoxon tests*.

c *χ^2^ tests*.

1 *TB correlated symptoms include cough, expectoration, fever, fatigue, hemoptysis, appetite loss, dyspnea, respiratory distress, insomnia, palpitation, weight loss*.

2 *Patients without typical TB symptoms; found and diagnosed by physical examination*.

*Bold numbers indicate a P-value of < 0.05*.

In patient groups with ages of 15–55 and 56–74, the ratios of men (37.2 and 39.4%) who were admitted to hospital within 1 month after onset of the symptoms were higher than those of women (24.7, 20.4%, respectively); no differences were found in this case between men and women patients older than 75.

**Figure 1:**
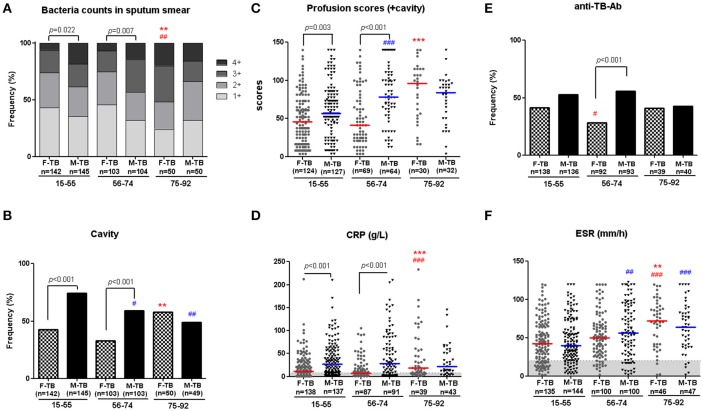
Age-associated male bias in active TB patients without treatment. Disparity on grades of sputum bacterial counts **(A)**, cavity **(B)**, weighted profusion scores **(C)**, and CRP levels **(D)** were found between M-TB_15−74_ and matched F-TB_15−74_, but not between the genders with ages older than 75. Results of anti-TB antibody response **(E)** and ESR **(F)** in men and women with TB at different age stages are also shown. The χ^2^ tests were used to compare variables displayed as percentages. Differences between groups of **C,D,F** were analyzed by Mann–Whitney tests. Horizontal lines represent median values. ^#^compared with F-TB_15−55_ (red mark) or M-TB_15−55_ (blue mark). ^#^*P* < 0.05, ^##^*P* < 0.01; ^###^*P* < 0.001. *Compared with F-TB_56−74_ (red mark) or M-TB_56−74_ (blue mark). **P < 0.01; ***P < 0.001.

In T-SPOT.TB tests, there were no statistical difference between men and women in all matched ages on both the positive ratios (Table 1) and the counts of the spots (Supplementary Figure 1). In an antibody test for *Mtb*. antigens (LAM and 38 kD), postmenopausal women showed periodical decrease in positive ratio in F-TB_56−74_ compared to the age–matched M-TB_56−74_ and F-TB_15−55_ (Figure 1), but no difference was found between men and women in results of these two tests of *Mtb*. infection at other stages of age.

### Male Bias in Lung Lesions Is Age-Associated

Higher sputum bacterial counts (3+ and 4+) were observed in men with TB at ages of 15 to 74 years (F-TB_15−55_ vs. M-TB_15−55_, 26.1 vs. 38.6%; F-TB_56−74_ vs. M-TB_56−74_, 25.3 vs. 43.3%), but not in patients after the age of 75 (F-TB_75−92_ vs. M-TB_75−92_, 52 vs. 34%) (Figure 1), as sputum bacterial counts significantly increased in gerontic aged women.

**Figure 2:**
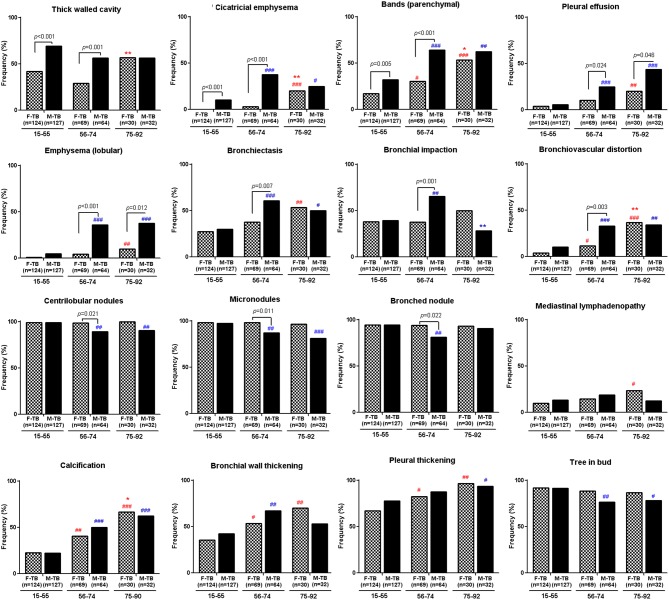
Differential age-associated changes in indices of CT images in men and women with TB. Data shown as positive ratios in each group. ^#^compared with F-TB_15−55_ (red mark) or M-TB_15−55_ (blue mark). The χ^2^ tests were used to compare variables displayed as percentages. ^#^*P* < 0.05, ^##^*P* < 0.01; ^###^*P* < 0.001. *Compared with F-TB_56−74_ (red mark) or M-TB_56−74_ (blue mark). *P < 0.05; **P < 0.01.

The analysis of the CT images revealed that both men and women with TB showed age-dependent increase in the injured zones of lung (Supplementary Figure 2) and in ratios of pleural thickening, pleural effusion, calcification and lobular emphysema (Figure 2). Aging-associated increase of bronchiectasis, bronchial wall thickening, cicatricial emphysema, parenchymal bands and bronchovascular distortion were only found in women with TB; cases with these indices decreased in M-TB_75−92_. Men with centrilobular nodules, micronodules, and tree in bud reduced after age of 55 (Figure 2).

Significantly higher ratios of cases with bronchiectasis, bronchial impaction, bullae, hilar lymphadenopathy and bronchovascular distortion with lower ratios of centrilobular nodules, micronodules, and bronched nodules were found in M-TB_56−74_ compared to the matched women while ratios of pleural effusion and lobular emphysema were significantly higher in men after the age of 55 than the matched women.

Consistent with the pattern of difference in sputum bacterial counts, the ratios of cavity (Figure 1), especially thick-walled cavity, and those of cicatricial emphysema, parenchymal bands (Figure 2) and weighted profusion scores were significantly higher only in M-TB_15−74_ than the matched women. The disparities of these indices were not found between men and women older than 75.

### Differential Critical Indices Associated With Cavity in Men and Women With TB

**Figure 3:**
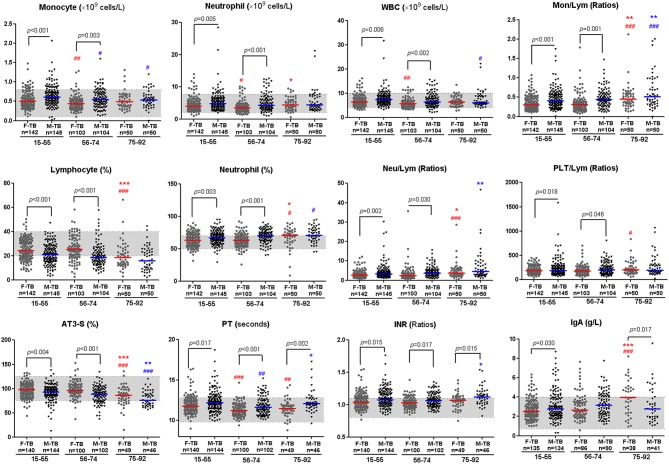
Inflammatory and coagulation indices with significant disparity between men and women with TB. Horizontal lines represent median values. Gray areas represent the normal ranges of the indices. WBC, white blood cells; Mon/Lym, monocyte to lymphocyte ratio; Neu/Lym, neutrophil to lymphocyte ratio; PLT/Lym, platelet to lymphocyte ratio; AT3, antithrombin III; PT, prothrombin time; INR, international normalized ratio; IgA, Immunoglobulin A. The differences between groups were analyzed by Mann–Whitney tests. ^#^Compared with F-TB_15−55_ (red mark) or M-TB_15−55_ (blue mark). ^#^*P* < 0.05, ^##^*P* < 0.01; ^###^*P* < 0.001. *Compared with F-TB_56−74_ (red mark) or M-TB_56−74_ (blue mark). *P < 0.05; **P < 0.01; ***P < 0.001.

Comparison of the clinical indices indicated that the age/sex-associated changes of C reactive protein (CRP), counts of neutrophils, monocytes and white blood cells (WBC), percentages of neutrophils and lymphocytes, ratios of Neu/Lym, Mon/Lym and PLT/Lym, and level of antithrombin III (AT3) were highly consistent with that of cavity and the sputum bacterial counts; the sex disparities of these indices were observed only in patients at the ages of 15-74 (Figure 3). The erythrocyte sedimentation rate (ESR) increased with aging in both men and women, especially in women after the age of 75, but no statistically significant difference of ESR was observed in all the paired groups (Figure 1).

The aging-associated changes and disparities of other indices between men and women with TB are shown in Figure 3, Supplementary Figures 1, 3, 4. Significant difference in coagulation-correlated prothrombin time (PT) and PT-associated international normalized ratio (INR) exists between men and women in the whole age span (Figure 3).

In multivariate logistic regression analysis including both men and women cases, male gender included in the model was highly associated with cavity with odds ratio (OR = 2.70). Higher age was associated with decreased cavity on the whole (OR = 0.5); while mean corpuscular hemoglobin concentration and ESR showed weak association (ORs were closed to 1) (Table 2). However, in a separate analysis, cavity in men was strongly and positively associated with complement component 4 (C4) (OR = 4092.06, *P* < 0.001) and negatively with BMI (OR = 0.84, *P* = 0.026). In women with TB, compared to the 15-55 age range, ages 56-74 were negatively associated with cavity (OR = 0.16), but ages 75-92 were positively associated with cavity (OR = 18.07). Positive response in anti-TB antibody test [TBAb(+)] (OR = 3.05, *P* = 0.023) had medium-strength association with cavity (Table 2).

**Table 2:** Indices associated with cavity in multivariate logistic regression analysis in men and women with TB separately and in combination.

**Groups**	***N***	**Indices in model**	**(95% CI)**			**Omnibus test** ***P***	**Correct ratio (%)**
			**OR**	**(Lower, Upper)**	***P***		
Men and women with TB	594	Age grade	0.50	(0.31, 0.79)	**0.003**	0.000	68.33
		MCHC (g/L)^a^	0.96	(0.93, 0.99)	**0.004**		
		ESR (mm/h)^a^	1.01	(1.00, 1.02)	**0.009**		
		Male^b^	2.70	(1.51, 4.85)	**0.001**		
Men with TB	299	BMI	0.84	(0.71, 0.98)	**0.026**	0.000	64.44
		FDP (g/L)^a^	0.91	(0.84, 0.99)	**0.031**		
		MCHC (g/L)^a^	0.95	(0.91, 0.99)	**0.009**		
		C4 (g/L)	4092.06	(35.87, 466880.64)	**0.001**		
Women with TB	295	Age_15−55_	Ref	Ref	**Ref**	0.000	69.52
		Age_56−74_	0.16	(0.04, 0.67)	**0.012**		
		Age_75−92_	18.07	(1.13, 289.28)	**0.041**		
		HGB (g/L)^a^	0.96	(0.93, 1.00)	**0.032**		
		ESR (mm/h)^a^	1.02	(1.00, 1.04)	**0.012**		
		TB Ab(+)^c^	3.05	(1.17, 7.97)	**0.023**		

*ORs are calculated from binary logistic analysis. Bold numbers indicate a P-value of < 0.05. MCHC, Mean corpuscular hemoglobin concentration; ESR, Erythrocyte sedimentation rate*;

*BMI, body mass index; FDP, fibrin and fibrinogen degradation product; HGB, hemoglobin; C4, complement component 3; TB-Ab, anti-TB IgG antibody*.

a *Indices with P < 0.05, but the value of OR ratio (95% CI) include /close to 1.000*.

b *Female as the reference*.

c *Patients with TB Ab (-) as the reference*.

Bold numbers indicate a P-value of < 0.05.

## Discussion

This study characterized the radiological and clinical indices in men and women with newly diagnosed TB, with a long age span from adolescent and adult to elderly ages. As we have excluded the cases with common confounding factors which bias the pathogenesis of TB (16, 17), the significant difference of lung lesions between men and women may reflect the overall impact of age-associated differential endocrines, especially sex hormones, sex-related genetic regulation, and metabolism on the host response to the infection.

On the whole, men with TB had higher ratios of cases presented with inflammatory/infection-associated indices either at ages of 15-74 (cavity, esp. thick-walled cavity, cicatricial emphysema, parenchymal bands), after age of 56 (pleural effusion, lobular emphysema), or periodically at 56-74 (bronchiectasis, bronchial impaction, bronchiovascular distortion, bullae, and hilar lymphadenopathy).

The accumulation of these detailed disparity results in the first intriguing finding that the male bias in TB pathogenesis is age-associated. The indices which reflect the severity of lung lesion on the whole directly (the ratios of cavity, especially thick-walled cavity), and indirectly (weighted profusion scores, sputum bacterial loads), as well as the typical indices which reflect inflammatory status (CRP, counts of WBC, neutrophil, monocytes, etc.) all were significantly higher in M-TB_15−74_ than in F-TB_15−74_, but not in men with TB older than 75.

It is intriguing that even the gender differences of time from onset of TB-associated symptoms to seeking healthcare in this study were consistent with the similar age-associated pattern of changes as mentioned above. A previous population-based survey which assessed health-seeking behavior in adults indicated a significant delay in women (41 vs. 19 days in men) with a cough of more than 3 weeks (18, 19). The author suggested that socio-economic and culture-associated sex inequalities may lead to poorer access to health care and delays to diagnosis of TB in women. However, this possibility has been excluded in a following survey based on large populations in Bangladesh (3). Our results from this and a previous study (20) with sputum smear positive patients may suggest a biological explanation of the age stage-associated delay of health-seeking behavior in women with TB: TB-induced lung lesions might be mild and less severe and therefore endurable in women compared to in men during adolescent and adult ages after onset of symptoms, while they become more severe in women than in men at elderly ages.

Age-associated changes in levels of sex steroid hormones may play key roles in this age-associated sex disparity in TB-induced lung lesions (10). In our study, two age points, 55 and 75, were set to divide the patients into three groups according to their age-associated differential levels of sex hormones. In general, at age 55, most women are postmenopausal; estradiol production in the ovaries ceases (21). Thereafter, only basal levels of progesterone are being synthetized by the adrenal glands. In aged women, dehydroepiandrosterone and testosterone levels decrease, yet follicle-stimulating hormone (FSH) and luteinizing hormone (LH) levels rise from the 4th decade onwards (22). In men, the concentration of serum testosterone starts declining steadily at ~30–40 years of age at a rate of about 1% per year (23); the level of free testosterone decreases by about 2–3% per year displaying no clear turning point (24). Accordingly, in men at ages of 75 and elder, the level of free testosterone will decline to about 1/3 of the level of the testosterone in men in their forties, although the average testosterone level remains within the normal range in most men. In turn, estradiol, estrone, LH, and FSH gradually increase (25).

In general, estradiol exhibits an enhancing effect, while testosterone exhibits an inhibitory effect on both the adaptive and innate immune systems (10). However, our data indicated that menopause in women did not change the bias of lung pathogenesis instantly, while the reducing cavities in aged men were seemly consistent with their theoretically decreasing testosterone levels. Moreover, low BMI in men but not in women was significantly correlated with cavity. As BMI and the associated obesity are regulated by androgen-estrogen balance (26, 27), the interplay between all the above-mentioned sex steroid hormones, rather than each isolated hormone, with aging immunity, may decide the differential extent of TB pathogenesis in men and women (28, 29).

Data from the WHO's annual report (1) (2017-annext II) compensate the limitation of our lacking data of TB with ages before puberty ( ≤ 14 years old): in most countries with high TB burden, remarkable sex bias in estimated TB incidence was found only in cases after puberty. The male/female ratios of estimated TB incidence in cases before 14 in these countries were mostly close to 1. Consistently, two analyses based on registering 31,358 new smear positive pediatric cases with TB in China (30), and on 10,744 patients in Tuscany (31) showed close notification ratios between boys and girls from the age of 0 to 14 years. These data further substantiated our hypothesis about the decisive role of sex hormones in sex disparity of TB pathogenesis.

We should emphasize, however, that this retrospective study was simplified by excluding the cases with confounding factors. In reality, although men and women non-smokers have similar proportions of LTBI in TB endemic area, according to a cross-sectional study in Taiwan (32), smoking may influence latent TB infection (LTBI) (32) and severity of active TB (33). Moreover, aging-associated chronic diseases, such as diabetes, hypertension, cardiac, hepatic or renal failure, chronic obstructive lung disease, and inflammatory arthritis, as well as medications, and even biological age-associated weight gain may change the levels of sex steroid hormones (34) and influence the susceptibility to TB (11). Stratified epidemiology research in the elderly by sex will reveal the overall influence of these factors on TB pathogenesis.

In addition, while male gender was identified as the only factor which is significantly associated with cavity in analysis of all 53 indices in all men and women patients, higher C4 level and positive anti-TB antibody response were identified as associated factors in men and women, respectively. Our data suggest that indiscriminate analysis of male and female cases may mask the key factors associated with pathogenesis. Very few studies have assessed the results of men and women separately (35); likewise, differential response to *Mtb*. infection and anti-TB treatment in either young or old men patients, and pre- or post-menopausal women patients have not yet been well-recognized. Therefore, our study emphasizes the importance of stratifying the analysis by both the sex and age in TB research to get an unbiased conclusion (36, 37).

Finally, as increasing levels of circulating antibody/antigen complexes with activation of classical complement (38, 39) characterized early disease in TB, understanding the influence of sex-associated endocrines on the complement and humoral immune response in TB progression (40) may help to reveal the mechanisms of the immune pathogenesis of TB in men and women. Further research is required to gain a better understanding of the differences in immunity to TB between men and women for future TB prevention and forecasting (41) at the community level (41, 42) and developing individualized treatment concepts for severe TB cases that take sex/age-specific host factors into account.

## Data Availability

All datasets generated for this study are included in the manuscript and/or the Supplementary Files.

## Ethics Statement

This study was conducted in accordance with the amended Declaration of Helsinki and the ethical guidelines of the institutional review board of Tongji University (Project approval number: K17-043). All participants gave written consent for the use of their clinical information for research purposes; written informed consent was obtained from the parents or guardians of participants under the age of 16. Clinical data were anonymized.

## Author Contributions

YF and JS designed research and wrote the article. JS and AS-L analyzed and interpreted radiological data. YC, FC, JH, GY, and XH collected clinical data. HY and WS interpreted the clinical data. YC, AS-L, and YF analyzed the data. All authors read and approved the final manuscript.

### Conflict of Interest Statement

The authors declare that the research was conducted in the absence of any commercial or financial relationships that could be construed as a potential conflict of interest.

## Supplementary Material

Supplementary Figure 1 Age-associated inflammatory and coagulation indices without significant disparity between matched men and women with TB. Horizontal lines represent median values. Gray areas represent the normal ranges of the indices. Each dot from T-SPOT counts represents the higher count from the two counts of the test for each case. RDW-SD, red blood cell distribution width Standard Deviation; C3, complement component 3; FDP, fibrin and fibrinogen degradation product; PCT, plateletcrit; IgG, Immunoglobulin G. The differences between groups were analyzed by Mann–Whitney tests. ^#^Compared with F-TB_15−55_ (red mark) or M-TB_15−55_ (blue mark). ^#^*P* < 0.05, ^##^*P* < 0.01; ^###^*P* < 0.001. ^*^Compared with F-TB_56−74_ (red mark) or M-TB_56−74_ (blue mark). ^*^*P* < 0.05; ^**^*P* < 0.01; ^***^*P* < 0.001.

Supplementary Figure 2 Injured zones and indices of CT images without significant disparity between men and women with TB. The χ^2^ tests were used to compare variables displayed as percentages. ^#^Compared with F-TB_15−55_ (red mark) or M-TB_15−55_ (blue mark). ^#^*P* < 0.05, ^##^*P* < 0.01; ^###^*P* < 0.001. ^*^Compared with F-TB_56−74_ (red mark) or M-TB_56−74_ (blue mark). ^*^*P* < 0.05.

Supplementary Figure 3 Age-associated change and disparity in transferrin, IgM and coagulation indices in men and women with TB. Horizontal lines represent median values. Gray areas represent the normal ranges of the indices. IgM, Immunoglobulin A; FIB, fibrinogen; C4, complement component 4; TT, thrombin time; PDW, platelet distribution width; MPV, mean platelet volume; P-LCR, platelet-large cell ratio; APTT, activated partial thromboplastin time. The differences between groups were analyzed by Mann–Whitney tests. ^#^Compared with F-TB_15−55_ (red mark) or M-TB_15−55_ (blue mark). ^#^*P* < 0.05, ^##^*P* < 0.01; ^###^*P* < 0.001. ^*^Compared with F-TB_56−74_ (red mark) or M-TB_56−74_ (blue mark). ^*^*P* < 0.05; ^**^*P* < 0.01.

Supplementary Figure 4 Age-associated changes of red blood cell indices between men and women with TB. Horizontal lines represent median values. Gray areas represent the normal ranges of the index in women, the area between dashed lines represents the normal ranges of the index in men. The differences between groups were analyzed by Mann-Whitney U tests. RBC, red blood cell; HGB, hemoglobin; HCT, hematocrit; MCH, mean corpuscular hemoglobin; MCV, erythrocyte mean corpuscular volume; MCHC, mean corpuscular hemoglobin concentration. The differences between groups were analyzed by Mann–Whitney tests. ^#^Compared with F-TB_15−55_ (red mark) or M-TB_15−55_ (blue mark). ^#^*P* < 0.05, ^##^*P* < 0.01; ^###^*P* < 0.001. ^*^Compared with F-TB_56−74_ (red mark) or M-TB_56−74_ (blue mark). ^*^*P* < 0.05; ^**^*P* < 0.01; ^***^*P* < 0.001.

## Abbreviations

AFB: acid-fast bacillus
BMI: body mass index
C4: complement component 4
CRP: C reactive protein
FIB: fibrinogen
HRCT: high resolution computed tomography
INR: international normalized ratios
OR: odds ratio
PT: prothrombin time
TB: tuberculosis
TB-Ab: anti-TB IgG antibody.
