# Supplementary figures and images for: Sex Disparity in Severity of Lung Lesions in Newly Identified Tuberculosis Is Age-Associated
Source: Front Med (Lausanne). 2019 Jul 17;6:163. doi: 10.3389/fmed.2019.00163 (PMC6650771; doi:10.3389/fmed.2019.00163)

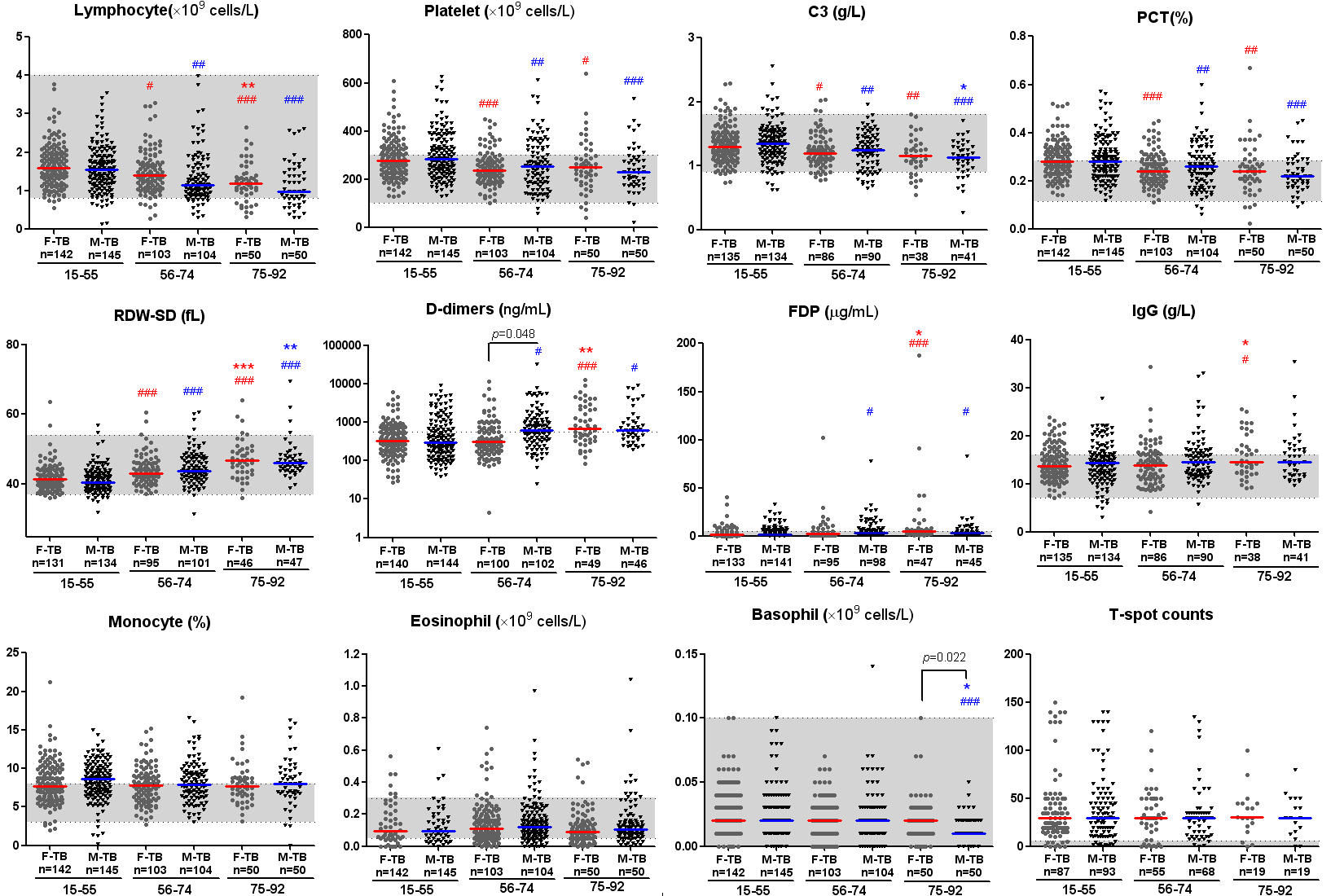

Supplement: Supplementary Figure 1 — Age-associated inflammatory and coagulation indices without significant disparity between matched men and women with TB. Horizontal lines represent median values. Gray areas represent the normal ranges of the indices. Each dot from T-SPOT counts represents the higher count from the two counts of the test for each case. RDW-SD, red blood cell distribution width Standard Deviation; C3, complement component 3; FDP, fibrin and fibrinogen degradation product; PCT, plateletcrit; IgG, Immunoglobulin G. The differences between groups were analyzed by Mann–Whitney tests. #Compared with F-TB15−55 (red mark) or M-TB15−55 (blue mark). #P < 0.05, ##P < 0.01; ###P < 0.001. *Compared with F-TB56−74 (red mark) or M-TB56−74 (blue mark). *P < 0.05; **P < 0.01; ***P < 0.001. [file Image_1.TIF]

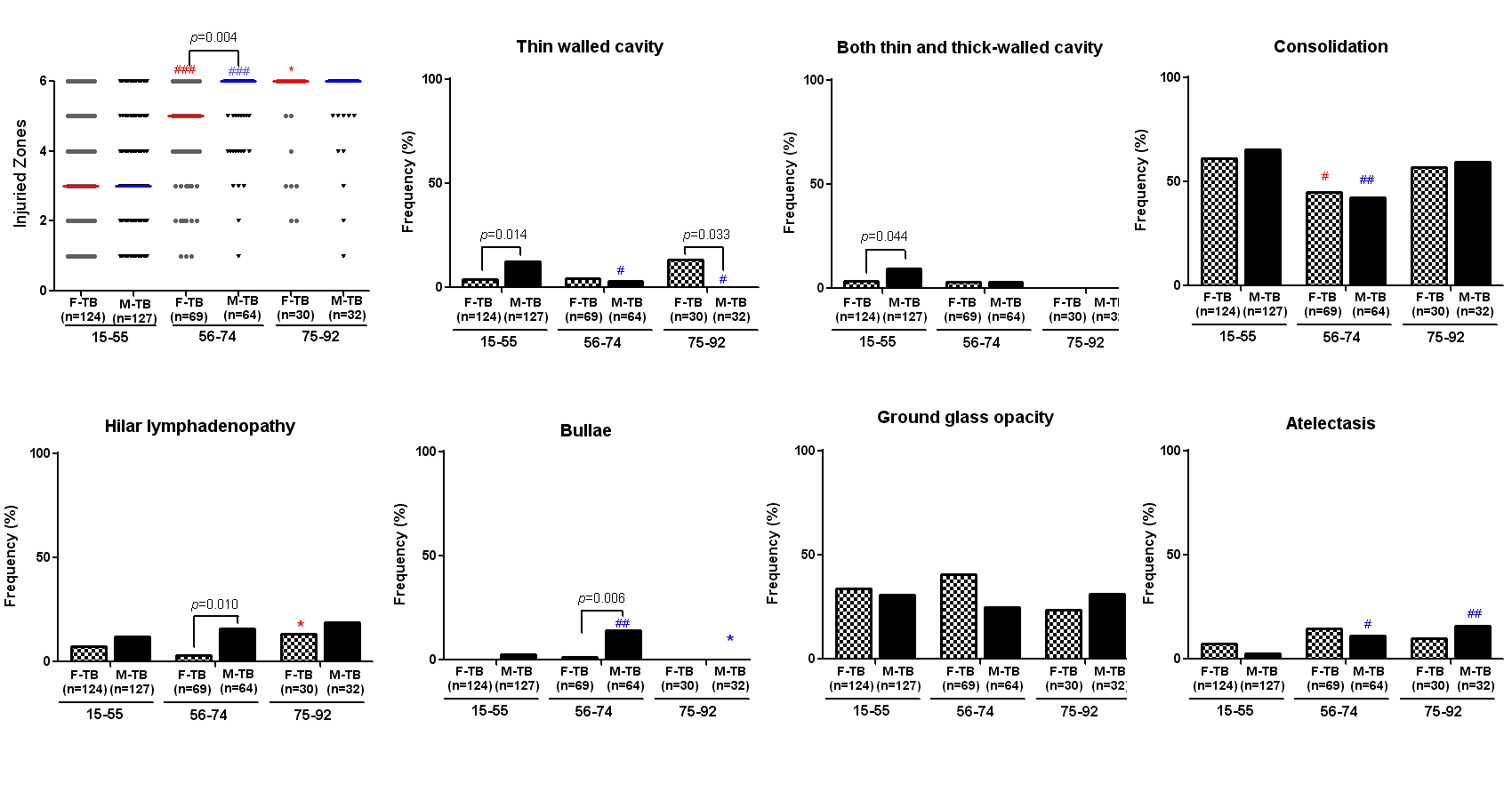

Supplement: Supplementary Figure 2 — Injured zones and indices of CT images without significant disparity between men and women with TB. The χ2 tests were used to compare variables displayed as percentages. #Compared with F-TB15−55 (red mark) or M-TB15−55 (blue mark). #P < 0.05, ##P < 0.01; ###P < 0.001. *Compared with F-TB56−74 (red mark) or M-TB56−74 (blue mark). *P < 0.05. [file Image_2.TIF]

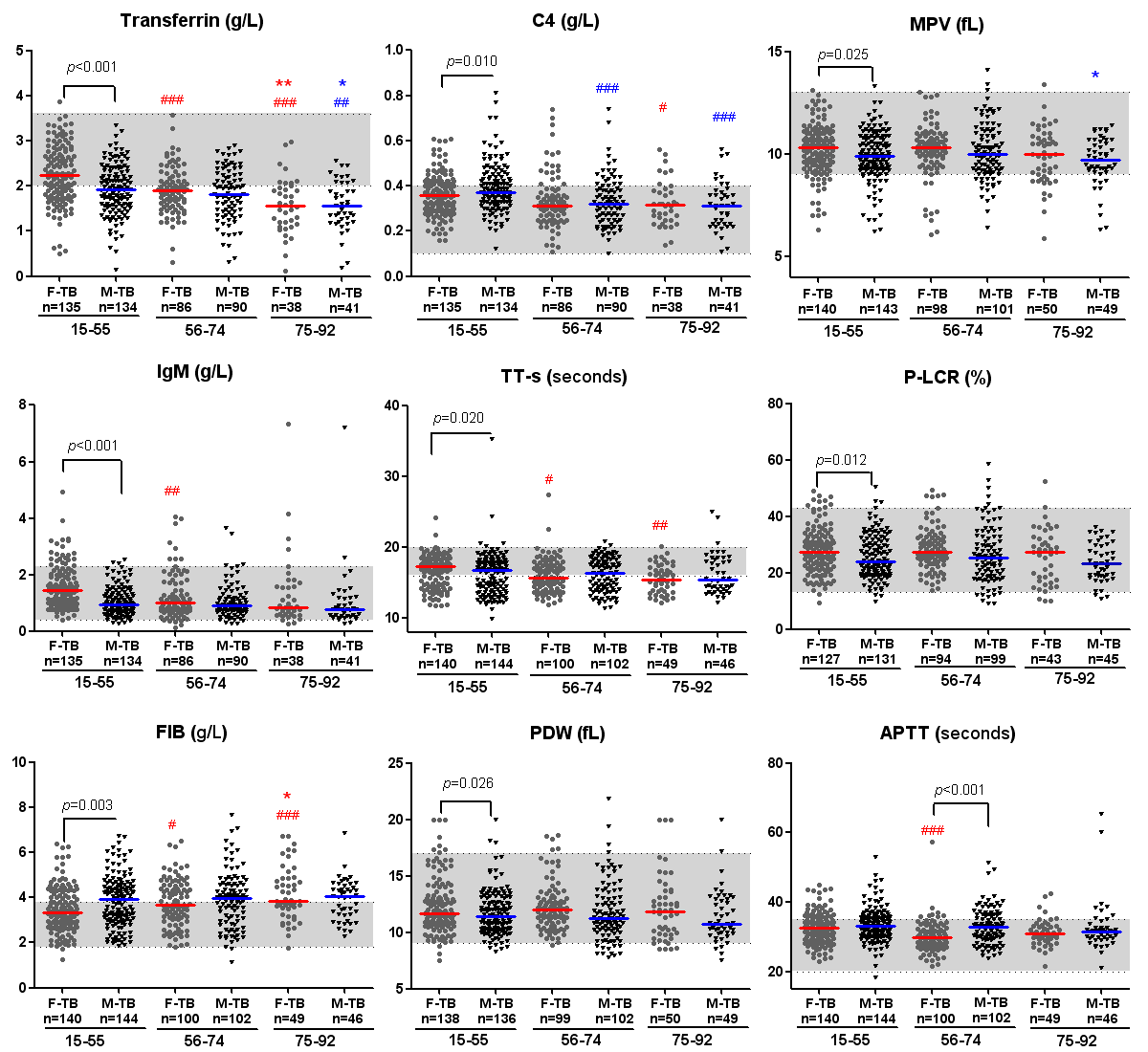

Supplement: Supplementary Figure 3 — Age-associated change and disparity in transferrin, IgM and coagulation indices in men and women with TB. Horizontal lines represent median values. Gray areas represent the normal ranges of the indices. IgM, Immunoglobulin A; FIB, fibrinogen; C4, complement component 4; TT, thrombin time; PDW, platelet distribution width; MPV, mean platelet volume; P-LCR, platelet-large cell ratio; APTT, activated partial thromboplastin time. The differences between groups were analyzed by Mann–Whitney tests. #Compared with F-TB15−55 (red mark) or M-TB15−55 (blue mark). #P < 0.05, ##P < 0.01; ###P < 0.001. *Compared with F-TB56−74 (red mark) or M-TB56−74 (blue mark). *P < 0.05; **P < 0.01. [file Image_3.TIF]

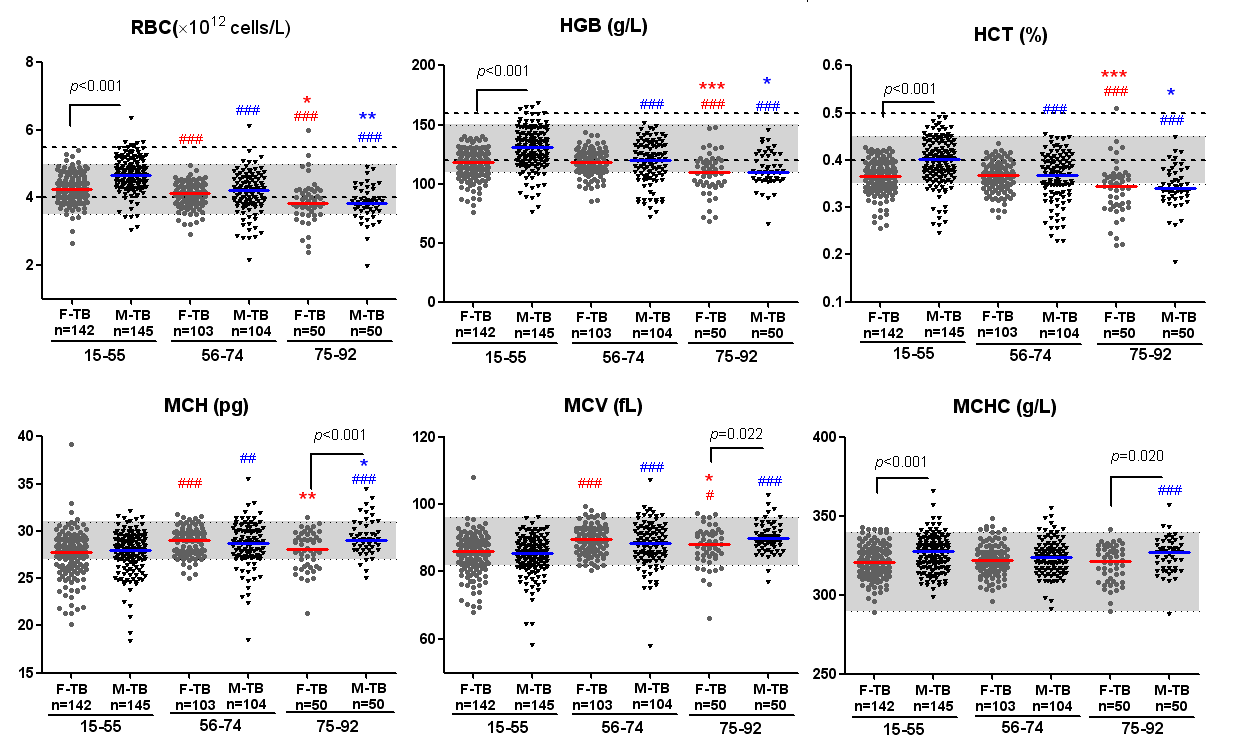

Supplement: Supplementary Figure 4 — Age-associated changes of red blood cell indices between men and women with TB. Horizontal lines represent median values. Gray areas represent the normal ranges of the index in women, the area between dashed lines represents the normal ranges of the index in men. The differences between groups were analyzed by Mann-Whitney U tests. RBC, red blood cell; HGB, hemoglobin; HCT, hematocrit; MCH, mean corpuscular hemoglobin; MCV, erythrocyte mean corpuscular volume; MCHC, mean corpuscular hemoglobin concentration. The differences between groups were analyzed by Mann–Whitney tests. #Compared with F-TB15−55 (red mark) or M-TB15−55 (blue mark). #P < 0.05, ##P < 0.01; ###P < 0.001. *Compared with F-TB56−74 (red mark) or M-TB56−74 (blue mark). *P < 0.05; **P < 0.01; ***P < 0.001. [file Image_4.TIF]
